# The Immune System of HIV-Exposed Uninfected Infants

**DOI:** 10.3389/fimmu.2016.00383

**Published:** 2016-09-28

**Authors:** Bahaa Abu-Raya, Tobias R. Kollmann, Arnaud Marchant, Duncan M. MacGillivray

**Affiliations:** ^1^Department of Pediatrics, Division of Infectious Diseases, University of British Columbia, Vancouver, BC, Canada; ^2^Institute for Medical Immunology, Université Libre de Bruxelles, Charleroi, Belgium; ^3^Department of Medicine, Dalhousie University, Saint John, NB, Canada

**Keywords:** HIV, HIV-exposed infants, innate immune system, cell-mediated immune system, humoral immune response

## Abstract

Infants born to human immunodeficiency virus (HIV) infected women are HIV-exposed but the majority remains uninfected [i.e., HIV-exposed uninfected (HEU)]. HEU infants suffer greater morbidity and mortality from infections compared to HIV-unexposed (HU) peers. The reason(s) for these worse outcomes are uncertain, but could be related to an altered immune system state. This review comprehensively summarizes the current literature investigating the adaptive and innate immune system of HEU infants. HEU infants have altered cell-mediated immunity, including impaired T-cell maturation with documented hypo- as well as hyper-responsiveness to T-cell activation. And although prevaccination vaccine-specific antibody levels are often lower in HEU than HU, most HEU infants mount adequate humoral immune response following primary vaccination with diphtheria toxoid, *haemophilus influenzae* type b, whole cell pertussis, measles, hepatitis B, tetanus toxoid, and pneumococcal conjugate vaccines. However, HEU infants are often found to have lower absolute neutrophil counts as compared to HU infants. On the other hand, an increase of innate immune cytokine production and expression of co-stimulatory markers has been noted in HEU infants, but this increase appears to be restricted to the first few weeks of life. The immune system of HEU children beyond infancy remains largely unexplored.

## Introduction

Mother-to-child transmission (MTCT) of human immunodeficiency virus (HIV) has been reduced from 14–48% (1) to less than 3% in both high-income countries (2–4) and resource-limited settings (5, 6). The vast majority of infants born to HIV-infected women, thus, are HIV-exposed uninfected (HEU). In countries with high prevalence of HIV infection, such as some parts of Sub-Saharan Africa, HEU infants can comprise a significant proportion reaching 30% of all infants (6).

The mortality rates of HEU infants are twice higher than HIV-unexposed (HU) peers in the second year of life in Zimbabwe (7), 4.6% through 4 months in Zambia (8), and 4.9% by 1 year in Africa (9). This relates in part to the elevated risk of HEU infants suffer from infectious disease; this is the case in both low- (10, 11) and high-income countries (12). Specifically, increased risk for contracting infections (13), higher risk for severity of infection (14–18), greater rates of hospitalization (15, 18), and mortality (7) have been documented in HEU as compared to HU infants. HEU infants appear particularly susceptible to invasive infection with Group B *Streptococcus* and *Streptococcus pneumoniae* invasive disease as well respiratory tract infections (14, 19, 20).

The underlying cause of the increased risk for infectious morbidity and mortality in HEU remains unknown, thus, can currently not be rectified. HEU infants have two unique exposures compared to their HU peers that have the potential to alter their developing immune system and with that potentially worsen their infectious disease outcomes: antiretroviral (ARV) drugs and maternal HIV infection (21). Some of the ARV drugs, such as zidovudine (ZDV), have mitochondrial toxicity likely due to inhibition of host cell gamma-polymerase and accumulation of somatic mitochondrial DNA mutations (22, 23), or due to direct interference with mitochondrial bioenergetics cascades (24, 25), and induction of reactive oxygen species formation leading to cell damage (26). *In vitro* studies have revealed that ZDV exposure inhibits hematopoietic progenitor cells, which may explain ARV’s associated decreased red blood cell, neutrophil, and lymphocyte counts (27, 28). ZDV also has the potential to impair the HEU infant’s innate immune system development (specifically granulocytes/macrophages) (27). Combination ARV therapy has been associated with larger and longer lasting suppressive effect on neonatal neutrophil and lymphocyte counts at age of 0–2 months as compared to ARV mono-therapy (28).

Even when the neonate escapes HIV infection, the HIV-infected maternal–fetus interface may present an altered environment for fetal growth and development. HIV-infected women are at increased risk for chorioamnionitis and deciduitis (29). Increased infection or inflammation of the uterine environment exposes the developing immune system of the neonate to antigens and a potentially pro-inflammatory milieu of cytokines and chemokines. It is also noteworthy that the vaginal microbiota appears to be altered in HIV-infected women (30), which may be of importance for early infancy colonization with microbes. The sum of these effects is conceptualized as an “active womb” of HIV-infected women that has the potential to prime and alter the development of the neonatal immune system.

We here review what is known about altered function (both adaptive and innate) during early life immune ontogeny of HEU infants.

## Adaptive Immune System of HEU Infants

### Cell-Mediated Immunity of HEU Infants

Previous studies described both the quantitative and functional measures of the cell-mediated immunity (CMI) of HEU infants. Data on the quantity and quality (function) of CMI among HEU infants are derived mainly from observational studies. Moreover, these studies are difficult to interpret and their results are inconsistent, challenging the ability to draw a definite conclusion. This is further complicated by variability of the cohort characteristics reported (age at enrollment, settings, ethnicity, time span of follow-up) and laboratory methodology (antigenic stimulus, functional test) utilized.

#### T-Cell Subsets of HEU Infants

The most reported immunological abnormality of HEU infants pertains to the frequency of immune cell subsets. Cluster of differentiation (CD) 4 T-cells have been relatively well studied in HEU infants, owing to both the vulnerability of CD4 T-cells to HIV infection and their important role as regulators of the immune system and acquired immunity. Lower CD4 T-cell counts (28, 31–37) and to a lesser extent lower CD8 T-cell counts (32, 33) have been reported in multiple studies contrasting HEU infants to HU peers. Maternal HIV viral load has been proposed as a correlate for subsequent HEU T-cells counts. At 2 and 6 months of age, HEU infants born to mothers with viral load >1000 copies/ml had lower CD4 T-cell counts compared to HEU infants born to mothers with viral load <50 copies/ml at the time of delivery (35). Decreased counts of circulating CD4 T-cells may limit antigenic coverage and subsequent response, eventually cumulating in increased severity of infections.

However, differences between HEU vs. HU T-cell counts may be more nuanced. It has been proposed that the difference in the quantity of circulating T-cells detected in HEU infants is in part due to difference in frequencies of subsets of CD4 T-cells. HEU newborns had lower CD4 to CD8 T-cell ratio, lower CD4 naïve, and CD8 naïve T-cell percentages, increased percentage of activated (CD8+ CD38^bright^) CD8 T-cells and memory (CD4+ CD45RO+) T-cells, augmented double-negative (CD3+ CD4− CD8−) and immature (CD4− CD8− CD5− CD44+) T-cells when compared to HU newborns (34). The elevated number of immature (CD4− CD8− CD5− CD44+) T-cells suggests that the physiologic thymic maturation pathway may be impaired in HEU children (34). In another study, activated (CD4+ HLA− DR+ CD38+) and memory (CD4+ CD45RA− RO+) T-cells were higher among HEU infants compared to infants born to HIV-uninfected women (38). The increase in the percentage of activated and memory T-cells and the diminished numbers of naïve T-cells suggests that this subset of lymphocytes had experienced antigenic exposure that could have resulted from either *in utero* exposure of the fetus to antigens or to non-antigen specific cell differentiation.

The altered numbers and subtypes of T-cells in HEU infants may or may not result in impaired T-cell immune function (21).

#### T-Cell Function of HEU Infants

Several studies have shown that HEU infants express altered CMI function and display impaired T-cell maturation. The CMI response of HEU infants has been studied primarily by measuring cytokine production and cell proliferation to both HIV-specific and non-HIV-specific antigens with reports of hyper- or hypo-activity depending on the stimulant (Table 1). It is proposed that increased CMI response in HEU infants may result from *in utero* exposure to HIV antigens and/or other antigens related to other infections in the HIV-infected mothers. By contrast, attenuation of the CMI response might be related to limited CD4 T-cells repertoire and progenitor cell dysfunction that might lead to T-cells with compromised function.

**Table 1:** **Function of cell-mediated immune system of HIV-exposed uninfected infants**.

Stimuli types	Main functional results
**HIV-specific**	
HIV antigens	**Cytokine production:**
	– Production of IL-2 by cord blood leukocytes (39, 40).
	**Cellular proliferation:**
	– No cellular proliferative response of cord blood mononuclear cells (41).
**Non-HIV specific**	
*Staphylococcus aureus* Cowan	**Cytokine production:**
	– Reduced cord blood mononuclear cell production of IL-12 when compared to infants born to HIV negative mothers (42).
PHA	**Cytokine production:**
	– Reduced IL-2 production when compared to HU infants (38).
BCG	**Cellular proliferation:**
	– No difference in proliferative response at 2 weeks when compared to HU infants (36). – Weaker proliferative response at 6–8 months (43) and at 10 weeks of life (36) when compared to HU infants. – Stronger proliferative response at 6 and 14 weeks of life (44) when compared to HU infants.
Anti-CD3 and anti-CD28	**Cellular proliferation:**
	– Higher proliferative response when compared to HU infants (41).
Staphylococcal enterotoxin B	**Cellular proliferation:**
	– Increased proliferative responses at 6 and 14 weeks of life when compared to HU infants (44)

*HIV, human immunodeficiency virus; IL, interleukin; IFN, interferon; CD, cluster of differentiation; PHA, phytohemagglutinin; BCG, Bacillus Calmette–Guérin; HU, HIV-unexposed*.

##### HIV-Specific T-Cell Response

It has been fairly well established that following exposure to HIV antigens, cytotoxic T lymphocyte responses can be elicited among HEU children aged 2–35 months (45–48). Following stimulation with HIV peptides, the production of interleukin (IL)-2 in mononuclear cells was also observed. Specifically, HEU cord blood leukocytes produced IL-2 following stimulation with HIV antigen in 40–42% of HEU infants (39, 40). However, limited data suggest that cord blood mononuclear cells of HEU newborns fail to proliferate in response to synthetic HIV envelope peptides (41). The clinical significance of these findings thus is currently unclear as IL-2 has the potential to promote the expansion and influence the differentiation of effector CD4 T-cells into different subtypes: Th1, Th2, Th17, and T regulatory cells (49). While the aforementioned studies (45–48) may suggest that HEU infants have protective CMI responses to HIV, functionally this may not translate into effective protection. CD4 T-cells from HEU infants that were infected *in vitro* with HIV had higher levels of p24 protein as compared to healthy controls (50). This suggests that CD4 T-cells of HEU infants could in fact be more susceptible to *in vitro* HIV infection as compared to healthy controls (50).

##### Non-HIV-Specific T-Cell Response

While HIV-specific responses are of interest when considering MTCT and immunity to HIV, more traditional investigation of immune function has relied on generic stimuli to elicit CMI responses. In response to *Staphylococcus aureus* Cowan stimulation, infants of HIV-infected mothers had reduced cord blood mononuclear cell production of IL-12, a key Th1-response supporting cytokine, when compared to infants born to HIV-negative mothers (42). This suggests a possible suppressive effect of HIV exposure on Th1 immunity. However, in response to another polyclonal stimulus [phytohemagglutinin (PHA)], reduced IL-2 has been observed among HEU infants (38) as compared to HU infants. In the latter study, a stronger production of IFN-γ to IL-10 (the ratio of PHA-stimulated IFN-γ to IL-10 production) of cord blood cells was observed among HEU than HIV-infected infants (51). No HIV-infected infant produced more IFN-γ than IL-10, while 43% of HIV-uninfected infants produced higher IFN-γ than IL-10 (51). It is important to note that these stimuli activated cells via different, and often poorly understood mechanisms (52–59).

Studies have used different methods, culture techniques, timing, and stimuli for assessing HEU T-cell proliferation with evidence of both hypo and hyper proliferation. Higher lymphoproliferative responses to polyclonal T-cell activators (anti-CD3 and anti-CD28) were observed in cell cultures from HEU neonates born to HIV-infected mothers with uncontrolled HIV replication compared to HEU neonates born to HIV-infected mothers with undetectable viral load and HU (41).

Other groups used different mitogenic stimuli and reported increased proliferative responses among HEU infants to staphylococcal enterotoxin B at 6 and 14 weeks of life (44), and to PHA at 6–8 months of life (43) as compared to HU infants. Assessment of HEU infant T-cell proliferative responses to BCG vaccination have provided discordant results, with no difference at 2 weeks (36), weaker response at 6–8 months (43) and at 10 weeks of life (36), and stronger response at both 6 and 14 weeks of life (44) when comparing HEU infants to HU controls.

#### Consistency and Controversy of Cell-Mediated Immunity Findings

In summary, the most consistent findings are that HEU infants have lower CD4 T-cell, lower CD8 T-cell counts, increased activated and memory T-cells as compared to infants born to HIV-uninfected women and there is compelling evidence that HEU infants have HIV-specific cellular memory response. HIV and associated antigenic exposure can take place during gestation. Data support that HIV can cross the placenta during gestation as HIV has been detected in fetal tissues (60). HIV exposure appears to be able to activate HIV-specific memory responses (45–48, 61). The HIV-specific T-cell immune response, at least in part, is suppressed by Treg cells (61). Depletion of Treg cells increased HIV-specific T-cell immune responses among HEU infants (61).

It should be noted that there is controversy regarding the T-cell response to non-HIV-specific stimulation. Mixed hyper- and/or hypo-responsiveness to T-cell activation and the different pro-inflammatory cytokines profile may stem from different experimental conditions, variable population characteristics, maternal HIV viral load, and ARV exposure.

If the HIV-infected *in utero* milieu leads to priming of the HEU immune system, it follows that this takes place in a complex manner. This may be a simplified explanation for the complex reality that leads to diversity in HEU hyper- vs. hypo-responsiveness that may be deemed immune dysregulation. It further stands to reason that there will be population variation in such immune dysregulation, which may explain both discordant results and how many HEU infants go on to lead relatively healthy lives compared to some of their peers. Ultimately, it is suspected that for some stimuli the afflicted HEU infant will be hyper-responsive, while being normal or hypo-responsive to other stimuli.

### Humoral Immune Response of HEU Infants to Primary Vaccination

The quantity and function of passively acquired maternal antibodies conveyed to HEU infants is reviewed in a companion article of this Research Topic by Abu Raya et al. (62). Primary vaccination during infancy is of critical importance for prevention of infectious morbidity and mortality. It should be noted that the immunogenicity of primary vaccination according to the Expanded Program on Immunization (EPI), a World Health Organization initiative to expand vaccination programs throughout the world, was also evaluated among HEU infants. The summarized literature on HEU response to vaccination can be found in the following section (Table 2).

**Table 2:** **Vaccine-induced antibody response in HIV-exposed uninfected infants**.

Vaccine	Main results
Hepatitis B	Similar HBsAg IgG levels as compared to HU infants following primary vaccination, and antibody levels continued to be equivalent through the first 2 years of life (63). The majority (>99%) of HEU infants developed sero-protective HBsAg IgG levels (64). The prevalence of non-responders to HBV among HEU infants is 6.7–11.5% (65–67) and comparable to the 5–10% of non-responders reported among healthy population
Tetanus toxoid	Similar anti-TT IgG levels as compared to HU infants following primary vaccination, with the majority of HEU infants developing anti-TT IgG seroprotective levels (63–65, 68)
Diphtheria toxoid	Similar anti-DT IgG levels as compared to HU infants following primary vaccination, and 95–99% of HEU infants developed anti-DT IgG sero-protective levels (64, 65)
Pneumococcal conjugate	Higher pneumococcal-specific antibody levels as compared to HU infants following the second (68) and third 7-valent PCV-doses (69)
*Haemophilus influenzae* type B	Similar anti-Hib IgG levels as compared with HU infants, following primary vaccination (64, 68) with 91–98% retaining protective anti-Hib IgG levels till 2 years of age (63, 70)
Whole cell pertussis	Higher anti-pertussis antibody levels as compared with HU infants following primary vaccination (63, 64, 68)
Measles	Comparable anti-measles antibody levels as compared with HU, following one and two measles vaccine doses (63, 71). The vast majority (99%) of HEU children had sero-protective antibody levels against measles at 7–15 years of age (72)

*HBsAg IgG, hepatitis B surface antigen immunoglobulin G; HEU, HIV-exposed uninfected; HU, HIV-\unexposed; HBV, hepatitis B vaccine; DTP, diphtheria, tetanus toxoid, cellular pertussis vaccines; TT, tetanus toxoid; DT, diphtheria toxoid; PCV, pneumococcal conjugate vaccines; Hib, Haemophilus influenzae type b*.

#### Hepatitis B Vaccine

Data on the immunogenicity and seroprotection provided to HEU infants following primary immunization with hepatitis B vaccine (HBV) suggest that the majority of HEU infants produce adequate humoral immune response.

In South Africa, prevaccination (at 2 and 6 weeks) hepatitis B surface antigen (HBsAg) IgG levels, presumed maternally derived, were significantly higher among HEU than HU infants, with one-quarter of HEU infants having sero-protective levels (HBsAg IgG > 10 mIU/ml) (63). Following primary vaccination according to the EPI, HBsAg IgG levels at 3 months (after two HBV doses given at 6 and 10 weeks of age) were similar between HEU and HU infants and continued to be equivalent through the first 2 years of life (63). A subsequent study of South African HEU infants reported that >99% of HEU infants developed sero-protective antibody levels (HBsAg IgG >10 mIU/ml) 1 month after completing the primary HBV series (64). This small South African series would suggest that HEU infants do indeed produce adequate acquired immunity in response to HBV vaccination.

The prevalence of non-responders to primary series of HBV was also described. Among a cohort of Brazilian HEU infants vaccinated with HBV (at birth, 1 and 6 months), 6.7% of HEU infants were non-responders to HBV (HBsAg IgG <10 mIU/ml) 1 month after the third dose of HBV (65). This study supports earlier findings which reported that about 8% of HEU infants did not respond to HBV (66) and more recent data from India which found that of HEU infants vaccinated with three doses of recombinant HBV (at 6, 10, and 14 weeks), 11.5% were non-responders 3 months after the third dose of the vaccine (67). The reported prevalence of non-responders to HBV among HEU infants, 6.7–11.5% (65–67) is comparable to the 5–10% of non-responders reported among healthy population (73).

In sum, though timing of vaccination and population characteristics are variable, it does appear that the majority of HEU infants are capable of mounting a sufficient immune response for HBV vaccination to confer adequate seroprotection. What remains to be determined is how resilient these responses are and whether they wane over time. There may also be a sub-population within HEU infants that are at higher risk and do not mount adequate immune responses to HBV vaccination.

#### Tetanus Toxoid Vaccine

Studies that evaluated the immunogenicity and seroprotection following tetanus toxoid (TT) vaccination reports that HEU infants develop robust immune response. One month following the completion of the vaccination series with diphtheria-tetanus toxoid-cellular pertussis-*Haemophilus influenzae* type b (DTP-Hib) (given at 2, 4, and 6 months) in Brazil, all HEU infants showed sero-protective immune response to TT (anti-TT IgG >0.1 IU/ml) (65). And following the completion of 3 DTP vaccines doses (at 6, 10, and 14 weeks) in South Africa, anti-TT IgG levels were similar between HU and HEU infants, and the majority of HEU developed sero-protective levels to TT (63, 64, 68). Lowest prevaccination anti-TT IgG levels were associated with greatest vaccine-induced immune responses at 16 weeks (68).

#### Diphtheria Toxoid Vaccine

Data also support that HEU infants mount a quantitative immune response to diphtheria toxoid (DT) vaccine that is comparable to HU infants. Specifically, Brazilian HEU infants vaccinated with DTP-Hib (at 2, 4, and 6 months) has been shown to have similar anti-DT IgG levels compared to infants not exposed to HIV, 1 month after the 3rd dose of the vaccine (65). Moreover, the percentage of non-responders to DT vaccine (anti-DT IgG <0.1 IU/ml) was not significantly different between the groups, with 94.7% of HEU subjects and 98.2% of HU infants, having protective levels against DT after completion of primary series of three DTP doses (65). These findings were supported by a study in South Africa that found antibody levels against DT to be similar between HEU and HU, 1 month following the completion of primary vaccination series with diphtheria-toxoid, tetanus-toxoid, whole cell pertussis-HibCV (DTwP-HibC) according to the EPI (at 6, 10, and 14 weeks) (64). In addition, a high proportion (>99%) of HEU children developed sero-protective levels to DT vaccine (anti-DT IgG ≥ to 0.01 and 0.1 IU/ml) (64).

Though limited to only two studies on the subject, it appears as though HEU infants are not significantly different from peers with respect to response to DT vaccine.

#### Pneumococcal Conjugate Vaccine

Evaluation of the immune response to 7-valent pneumococcal conjugate vaccine (PCV) showed higher pneumococcal-specific antibody levels following the second (68) and third 7-valent PCV-doses (69) in HEU infants as compared to HU controls. Specifically, Jones et al. found that among a cohort of South African HEU infants vaccinated with two doses of 7-valent PCV (at 6 and 14 weeks), HEU infants had higher levels of pneumococcal-specific antibody levels than HU at 16 weeks of age (68). Moreover, infants with the lowest prevaccination pneumococcal-specific antibody levels had the most robust immune responses to the pneumococcal vaccine at 16 weeks (68). However, the clinical significance of these robust results is complicated by lack of well-established correlate of protection for collective antibody response to pneumococcal vaccine (68).

The largest study to date to report the immune response following primary PCV immunization in HEU infants described a cohort of HEU infants in South Africa (69). Prevaccination (age <6 weeks), HEU infants had lower titers and lower proportion of children with serotype-specific anti-capsular antibody ≥0.35 μg/ml to all serotypes (4, 6B, 9V, 14, 18c, 19F, and 23 F) compared to HU infants (69). At 10 weeks, antibody levels following the first 7-valent PCV-dose (given at age 6 weeks) were lower for four serotypes (6B, 14, 19F, and 23F) and the proportion of children who had serotype-specific anti-capsular antibody ≥0.35 μg/ml were lower for most serotypes (6B, 9V, 14, 19F, and 23F) in HEU as compared to HU infants (69). Following the second 7-valent PCV-dose (at 10 weeks), the antibody levels measured at 14 weeks of age were similar in HEU and HU infants for the majority of serotypes (6B, 9V, 14, 18c, 19F, and 23 F) (69). Three to six weeks following the third 7-valent PCV-dose (given at 14 weeks), the serotype-specific anti-capsular antibody levels were higher among HEU infants as compared to HU infants for all serotypes (69). When integrated, these data suggest that a two-dose primary series schedule in HEU children may afford similar protection against vaccine serotype-specific invasive pneumococcal disease as compared to HU children. However, it is noteworthy that the functionality of anti-pneumococcal antibodies as a correlate of protection is not well founded, and when assessed for opsonophagocytic activity there was an absence of consistency in the pattern between HEU and HU infants (69).

#### *Haemophilus influenzae* Type b Vaccine

Current data on the immunogenicity of *Haemophilus influenzae* type b (Hib) vaccine administered to HEU infants is reassuring as HEU infants vaccinated according to the EPI mounted robust and immune responses that were similar to HU infants following vaccination with Hib vaccine.

Within 1 month following the completion of primary immunization (6, 10, and 14 weeks), South African HEU infants had similar anti-Hib antibody levels as compared with HU at the age of 16 weeks (64, 68). Gaensbauer et al. reported findings from a cohort of Ugandan HEU infants vaccinated with Hib polysaccharide conjugate vaccine at 6, 10, and 14 weeks of age. By 12 weeks of age (following two Hib vaccine doses), 91% of HEU had protective levels (anti-Hib IgG >1.0 μg/ml), as did all infants by 24 weeks after three doses. Interestingly, maternally derived anti-Hib IgG levels at birth did not correlate with anti-Hib IgG levels at 12 or 24 weeks. At 48 weeks, the vast majority (98.3%) of HEU still retained protective levels (70).

The duration of HEU infant immune response to primary Hib vaccination has been derived from longitudinal studies. Data from a South African cohort showed that although prevaccination anti-Hib IgG levels (at 2 and 6 weeks) were lower among HEU compared to HU infants, antibody levels were similar between both groups at 3 months (after two doses given at 6 and 10 weeks) and until 2 years of age (63).

Thus, current data suggest that HEU infants mount immune response to Hib vaccine similar to HU infants that lasted until 2 years of age.

#### Pertussis Vaccine

Several studies have demonstrated that HEU infants mount higher immune response to primary vaccination with whole cell pertussis vaccine (given according to the EPI) compared to HU infants (63, 64, 68). Jones et al. reported data from a South African cohort and found that despite having lower anti-pertussis titers at birth, HEU infants had higher anti-pertussis antibody levels compared to HU 2 weeks following the completion of primary vaccination with thee doses of DTP-Hib vaccine (at 6, 10, and 14 weeks) (68). Infants with the lowest prevaccination anti-pertussis levels exhibited the greatest vaccine responses at 16 weeks (68). Reikie et al. has found that at 3 months, anti-pertussis IgG levels were similar between South African cohort of HEU and HU infants after two doses of DTP vaccine (at 6, 10 weeks), and higher for HEU as compared to HU at the age of 6 and 12 months (63). Moreover, Simani et al. reported that 1 month following the primary DTP-HibC vaccine series (at 6, 10, and 14 weeks) the anti-pertussis-toxoid titers were higher in HEU than in HU infants (64). It should be noted that the aforementioned studies used whole cell pertussis vaccine as compared to acellular pertussis vaccine that is currently employed by most developed countries. Moreover, the read out measure is critical as measuring collective antibody response to multiple antigens (63, 68) might be less accurate than antigen-specific responses. The lack of well-established sero-protective antibody levels is another challenge for extrapolating these finding to clinical settings. Overall, though more data are required on acellular pertussis vaccination among HEU infants, it does appear as though HEU infants mount satisfactory responses to whole cell pertussis vaccination.

#### Measles, Mumps, and Rubella Vaccine

Measles vaccine generated both comparable (63) and lower immune response (71) among HEU infants as compared to HU infants.

The proportion of South African HEU infants with sero-protective titers against measles was similar compared with HU infants, 28 weeks following the first measles vaccine dose (administered at nearly 40 weeks of age) and 2 weeks after the booster dose (administered at nearly 68 weeks of age) (71). However, waning immunity in HEU was noticed 9.5 months post booster, when HU had higher anti-measles titers and were more likely to have seroprotective titers (94.3%) compared with HEU (79.6%) infants, bringing the durability of HEU immune response into question (71). In contrast to the previous findings, the average levels of anti-measles virus antibodies were nearly indistinguishable between South African HEU and HU infants prevaccination (at 2 and 6 weeks), at 12 months (post 1^st^ vaccine dose given at age of 9 months) and at 2 years (post 2^nd^ vaccine dose given at 18 months) (63).

The long-term vaccine-induced immune memory against measles, rubella, and mumps was recently explored (72). A large US cohort of HEU children was evaluated at the age of 7–15 years, among which 87% of HEU infants had received two doses of measles, mumps, and rubella vaccine. The prevalence of measles seroprotection, rubella seroprotection, and mumps seropositivity were 99, 98, and 97%, respectively (72). Thus, although limited to one study, it appears that HEU infants retain long-term immunity to the measles, mumps, and rubella vaccine.

## Innate Immune System of HEU Infants

The innate immune system is the immediate, first line of defense against infectious agents that react rapidly upon encountering a pathogen. It is composed of different cells, including neutrophils, dendritic cells (DCs), monocytes, and natural killer (NK) cells.

In the 1980s, *in vitro* data showed that the development of the innate immune system can be impacted by ARVs as ZDV has been shown to suppress hematopoietic stem cells progenitors of granulocyte and macrophages (27). Several studies followed and evaluated the quantity and the function of innate immune system cells of HEU infants (Table 3).

**Table 3:** **Quantity and function of innate immune system of HIV-exposed uninfected infants**.

Innate immune cell	Count and/or function
**Neutrophils**	**Count:**
	– Lower ANCs in HIV/ARV-exposed infants as compared to HIV and ARV unexposed infants at 5 weeks and 8 months of age (74). – Lower ANCs were associated with ARVs exposure in HEU children till 8 years of life (33, 75).
**Monocytes**	**Count:**
	– Lower monocyte counts in HIV/ARV-exposed infants compared to HIV and ARV unexposed infants at 5 weeks of age (74). – Similar proportions of monocytes in HEU as compared to HU infants during the first year of life (76).
	**Function:**
	– Enhanced pro-inflammatory response of monocytes to bacterial ligand stimulation up to 6 weeks of age in HEU as compared to HU infants (76).
**Dendritic cells**	**Count:**
	– Higher percentage of mDCs in HEU as compared to HU newborns at birth but similar proportion of mDCS and pDCs during the first 3–12 months (77) and during the first year of life (76).
	**Function:**
	– Higher responsiveness of mDCs to stimulation with LPS at birth, as compared to HU (77) – Enhanced pro-inflammatory response of mDCs to bacterial ligand stimulation up to 6 weeks of age, as compared to HU (76). – Similar response of pDCs to stimulation with CpG ODN and similar IFN-α production by pDCs in HEU and HU infants at birth (77).
**NK cells**	**Count:**
	– No difference in the percentage of total NK cells and the percentage of different NK cell subsets HEU and HU infants at birth (78).
	**Function:**
	– Decline in the percentage of activated and perforin positive NK cells among HEU infants over the first year of life (78).

*ANC, absolute neutrophil counts; HIV, human immunodeficiency virus; ARV, antiretroviral; HEU, HIV-exposed uninfected; HU, HIV-Unexposed; ZDV, zidovudine; DCs, dendritic cells; mDCs, myeloid dendritic cells; pDCs, plasmacytoid dendritic cells; LPS: lipopolysaccharide; CpG ODN, CpG oligodeoxynucleotides; IFN-α, interferon-α*.

### Neutrophils

HIV and ARVs exposed infants have lower neutrophil counts than HIV and ARVs unexposed infants (74). Data suggest that suppression of hematopoietic stem cell progenitors is the most plausible mechanism as reduced neutrophil levels have been shown to be non-immune mediated (74) and ARV exposure has been shown to affect two other lineages (platelets and lymphocytes) (79, 80).

The dynamics of neutrophil counts over time was described. In HIV and ARV-exposed infants (90/92 confirmed HEU), neutrophil levels were lower than in HIV and ARV unexposed infants from birth until 20 months of age, reaching statistical significance at 5 weeks and 8 months (74). In another study, neutrophil levels of HEU subjects, although not compared to HU controls, were high shortly after birth, declining to reach a nadir at 2 months, then increasing until approximately 7 months; after this, the increase was more gradual before stabilizing at 3.5 years (75).

Exposure to ARVs is associated with decrease in neutrophil levels lasting till 18 months of age (33) and up to 8 years of age in another study (75). Thus, it appears that ARV exposure have a long-term effect on neutrophil counts. However, whether this long-term effect is affected by the timing of ARV exposure (*in utero*, neonatal or both) has not been established yet.

### Dendritic Cells

Circulating DCs are human antigen-presenting cells (APC) and are the precursors of tissue and lymphoid organ DCs; conventional DCs [cDCs, also known as myeloid DCs (mDCs)] and plasmacytoid DC (pDC) are the two main subsets of human DCs. Both cell types mediate the downstream immune response based on the cytokines they secrete (81).

Few studies described the frequency and function of DCs among HEU infants. HEU newborns are reported as having a higher percentage of mDCs than HU newborns at birth in one study (77), but all other currently available data suggest that HEU and HU infants have similar proportion of cDCS and pDCs during the first 3–12 months (77) and during the first year of life (76). It must be noted that these reports were based on proportion rather than absolute count; thus, it is at this time inconclusive whether DCs counts are affected by perinatal ARV and HIV exposure.

Data on the function of DCs among HEU infants are also limited, and is based on reports of *in vitro* study of TLR agonists and cytokine responses.

At birth, mDCs of HEU infants exhibited higher responsiveness to stimulation with LPS (via TLR4) than HU (77). As compared to HU, a greater proportion of HEU mDCs responded to bacterial ligand stimulation (PAM via TLR1/2 and LPS via TLR4) by secretion of pro-inflammatory cytokines (TNF-alpha, IL-6, IL-12) up to 6 weeks of age (76). Beyond 6 weeks and until 1 year of age, the response of mDCs to bacterial ligands was comparable between HEU and HU infants (76).

At birth, pDCs of HEU and HU responded similarly to CpG ODN stimulation (via TLR9) (77) and secreted similar levels of IFN-α, indicating a normal stimulated pDC function (77). In another study, a comparable proportion of pDCs that responded to bacterial ligands by secretion of inflammatory cytokines were reported among HEU and HU infants until 1 year of age (76).

Taken together, existing data suggest that gestational HIV-1 exposure increases HEU infants’ mDCs responsiveness at birth and enhances a pro-inflammatory response to bacterial ligand stimulation up to 6 weeks of life that normalizes by 1 year of age. This is limited to only a pair of studies and the clinical significance of these findings is yet unknown.

### Monocytes

Limited data exists on the quantity and function of monocytes among HEU infants. Monocyte counts were lower in the HIV- and ARV-exposed infants than in unexposed controls from birth until 8 months of age, reaching statistical significance at 5 weeks of age (74). However, in another study, the proportions of monocytes in HEU as compared to HU infants were not statistically different during the first year of life (76). The difference in absolute levels (74) of monocytes might have stronger clinical implications that the reported proportion (76).

Monocyte activity has been assayed though whole blood response to different TLR ligands, and has revealed differences between HEU and HU monocyte cytokine production during only the first 6 months of life (71). Specifically, a higher proportion monocytes from HEU infants mounted pro-inflammatory cytokine response (TNF-α, IL-6, IL-12) to bacterial ligands (PAM via TLR1/2 and LPS via TLR4) as compared to HU (76). Conversely, HU infants’ monocytes responded more strongly to viral ligand stimulation (pI:C and R848) at 6 months. By 1 year of age, the differences in the innate immune system activation between HEU and HU monocytes completely disappeared (76). This study suggest that HEU monocytes may be affected in function, leading to stronger immune response toward bacterial threats at the cost of weaker viral responses during early life, yet this stands to be repeated and tested further.

### NK Cells

Natural killer cells are directly cytotoxic, often to virally infected cells, and do not require guidance by other cells. Currently, there is very limited data on the quantity and function of NK cells among HEU as compared to HU infants. At birth, the percentage of total NK cells and the percentage of different NK cells subsets (CD56 ^bright^, CD56+ CD16+, CD56− CD16+) was not significantly different between HEU and HU infants. The total NK cell percentage did not change significantly in both HEU and HU over the first year of life (78).

Functional comparison demonstrated a significant decline in the percentage of activated NK cells (CD56^dim^ subset) and perforin positive NK cells among HEU infants over the first year of life (78). By contrast, over time HU infants demonstrated an increase in perforin positive NK cells, a marker of cytolytic potential (78). The poor expression of perforin among HEU infants might have clinical implications as it might compromise the ability of HEU infants to control viral infection (78).

## Concluding Remarks

Current evidence suggests that during early life, the developing immune system of HEU infants differs from HU peers. The etiology of this altered immune ontogeny appears to be multifactorial, including *in utero* exposure to ARVs, HIV antigens and/or other antigens related to other infections in HIV-infected mothers. Few longitudinal studies describe their dynamics over time. Although HEU infants are at increased risk for infectious disease morbidity and mortality, their quantitative humoral immune response to primary vaccination is adequate. This suggests that other mechanisms, including impaired qualitative antibody response, altered CMI, impaired innate immunity or infections caused by pathogens other than those covered by existing vaccines may be at the heart of increased morbidity and mortality of HEU infants. Transient innate immune system activation leading to enhanced pro-inflammatory response to bacterial ligands stimulation or immune tolerance to viral ligands has been reported among HEU infants. The origins of this innate immune system activation might stem from increased infection or inflammation of the uterine environment that exposes the developing immune system of the neonate with antigen and a potentially pro-inflammatory milieu. This has the potential to prime the neonatal immune system *in utero*. The idea of priming of the innate immune system is supported by recent evidence on memory of the innate immune system (82). Whether the postnatal differential pathogen-specific innate immune cells activation affects the susceptibility of HEU infants to severe infections should be further investigated.

Synthesizing a clear model for the changes of the developing immune system is challenging as studies have enrolled different HEU population at different ages; HEU subjects were exposed to different maternal viral loads and ARV regimens; the laboratory methods employed, the experimental conditions and the read out measurements were also different among studies. These studies still do have merit, as they do suggest that the perinatal environment may lead to an altered immune system.

## Author Contributions

BA, TK, DM, and AM searched the scientific literature; BA and DM wrote the first draft; BA, TK, DM, and AM reviewed and edited the manuscript; BA, TK, DM, and AM approved the final version.

## Conflict of Interest Statement

The authors declare that the research was conducted in the absence of any commercial or financial relationships that could be construed as a potential conflict of interest.
